# Revisiting Virchow’s Node: Exploring the Diagnostic Spectrum of the Supraclavicular Lymph Node Through Fine-Needle Aspiration Cytology in a Tertiary Care Hospital

**DOI:** 10.5146/tjpath.2025.13817

**Published:** 2025-05-31

**Authors:** Sumaira Qayoom, Nida Shabbir, Mala Sagar, Riddhi Jaiswal, Naseem Akhtar, Madhu Kumar

**Affiliations:** Department of Pathology, King George Medical University, Uttar Pradesh, India; Department of Surgical Oncology, King George Medical University, Uttar Pradesh, India

**Keywords:** Fine-needle aspiration cytology, Supraclavicular lymph node, Virchow’s node, Metastasis

## Abstract

***Objective: *** Virchow’s node, described in 1848, represents a metastasis to the left supraclavicular lymph node, commonly arising from gastric cancer. However, in regions with lower gastric cancer incidence, the relevance of Virchow’s node and the spectrum of diagnosis associated with supraclavicular lymphadenopathy (SCLAP) needs reevaluation. This study aimed to analyze the spectrum of fine-needle aspiration cytology (FNAC) at a tertiary care institute.

***Material and Methods:*** We retrospectively reviewed all supraclavicular lymph node aspirations performed between March 2019 and August 2022. Data were collected from the Department of Pathology’s electronic records and descriptive analyses were performed.

***Results: *** Out of 270 FNAC procedures for SCLAP, 50 were non-diagnostic. Of the 220 patients, cytological diagnosis was categorized as metastatic malignancy in 120 (54.5%) patients, granulomatous lymphadenitis in 57 (25.9%), reactive lymphadenitis in 11 (5.0%), acute suppurative lymphadenitis in 21 (9.5%), and lymphoproliferative disorder in 10 (4.54%) patients. Among the 120 metastatic cases, the most common type was adenocarcinoma (58.3%). The most common primary site was the lung (22.5%), oral cavity (19.2%), breast (12.5%), and gallbladder (10%). Primary gut carcinomas constitute only 9% of supraclavicular lymph node metastases.

***Conclusion:*** The findings suggest a need to reconsider the clinical significance of Virchow’s node, especially in regions with different cancer epidemiology. FNAC remains a critical diagnostic tool in evaluating SCLAP.

## INTRODUCTION

Virchow’s node was described in 1848 as a metastasis to the left supraclavicular lymph node, often from gastric cancer, which was later modified as the Virchow-Troisier node, as metastasis can occur from other abdominal organs. The supraclavicular lymph node is easily palpable and a favorable site for metastasis, hence also known as “Sentinel Node” (1). Fine needle aspiration cytology (FNAC) is efficient in diagnosing the etiology of enlarged lymph nodes and plays a significant role in evaluating neck lymph nodes, especially those with metastasis (2,3). FNAC confirms the presence of metastatic disease, and provides a clue regarding the nature and origin of the primary malignancy. Hence, it is an important and reliable tool for the follow-up of malignant conditions. Although supraclavicular masses are usually considered malignant, some studies have shown that lymph node enlargement can be secondary to various diseases, including inflammatory conditions. Contrary to this original idea, various studies have shown that gastric cancer is not a common primary cancer. Results have shown that enlarged supraclavicular lymph nodes do not point to the specific anatomic location but represent metastasis from common cancers in that particular population (4). With the dramatic changes in the epidemiology, screening, and management of cancers, one must consider whether “Virchow’s node” still holds the same clinical significance. This significance could be further diluted by the fact that gastric cancers are not common in our region, and infectious conditions such as tuberculosis have a high incidence. Therefore, this study aimed to analyze the diagnostic spectrum of supraclavicular lymph nodes in a tertiary care hospital using needle aspiration cytology.

## MATERIAL and METHODS

A retrospective review of all supraclavicular lymph node FNA procedures performed between March 2019 and August 2022 was done after obtaining ethical approval from the institutional review board. Demographic and clinical details were obtained from records in the Department of Pathology and were available on the electronic hospital information system. Only descriptive analyses were performed after data were anonymized. As a routine practice in our department, after explaining the procedure to the patient under aseptic conditions, FNA was performed using a 22 – 23 G needle and a 20 ml disposable syringe. Smears were stained with Hematoxylin & Eosin, MGG, and Papanicolaou stain. Wherever indicated, Ziehl–Neelsen staining for acid-fast bacilli was performed on an air-dried smear. Slides were collected from departmental archives and reviewed. Clinical data were analyzed, and descriptive statistics were used in the study. Statistical analyses were performed using anonymized Excel files in the Windows Statistical Package. For qualitative and quantitative variables, we conducted a descriptive analysis using percentages and means. P-Value <0.05 was considered as significant.

## RESULTS

**Figure 1:**
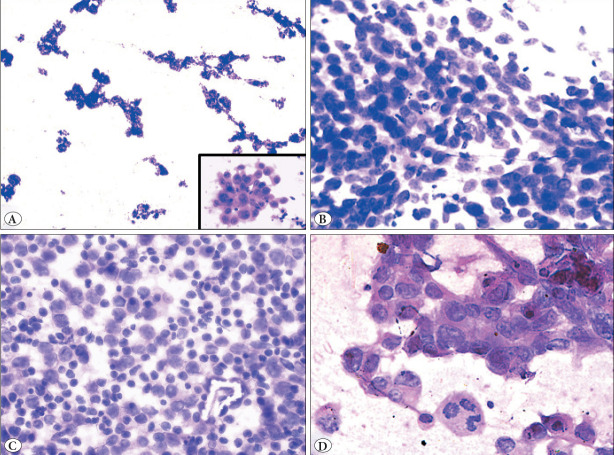
**A)** Smear shows Papillary fragments and acini formation (Inset) in a supraclavicular aspirate of known lung primary case (H&E 10X), **B)** Squamous cell carcinoma metastasis to left supraclavicular node in an operated case of carcinoma tongue (H&E 20X), **C)** Case of Non-Hodgkin Lymphoma presenting with left supraclavicular lymph node (H&E 20X), **D)** Metastasis of high-grade urothelial carcinoma to right supraclavicular lymph node in a 60-year-old male (H&E 40X).

FNA from the SCLN was performed in 270 patients, of which 50 were non-diagnostic and were excluded from the analysis. Of the remaining 220 patients, 123 (56%) were left-sided, 95 (43.1%) were right-sided, and two (0.9%) were bilateral. The study showed a female predilection in 132 (60%) patients and 88 (40%) male patients (Table 1). On cytological examination, 130 (59%) showed malignancy, 57 (25.9%) showed tuberculous lymphadenitis, and 11(5%) showed reactive lymphadenitis. A total of 22 (10%) cases were diagnosed as inflammatory or abscesses without any specific etiology (Table 2). Of the 130 malignant cases, 96 were observed in patients aged >40 years and 34 were in the < 40 years age group. Similarly, out of 90 benign cases, only 21 were observed in the age group >40 years, whereas 69 were observed in the younger age group (<40 years). This was statistically significant, with a p-value of < 0.00001. A total of 123 cases were observed on the left side, of which 84 (68%) were malignant, whereas 95 presented with right-sided SCLAP, of which 45 (47%) were malignant. This difference was statistically significant (p= 0.02). The most common histological type of malignancy was metastatic adenocarcinoma, seen in 70 (53.8%) cases, followed by metastatic squamous cell carcinoma in 28 (21.5%), lymphoproliferative disorder in 10 (7.6%), poorly differentiated/unclassified malignancy in 16 (12.3%), metastatic germ cell tumor in 3 (2.3), and urothelial carcinoma, small cell carcinoma, and malignant round cell tumor each in one case (0.8%) (Figure 1). Excluding lymphoproliferative disorders, metastasis constituted 54.5% (120/220) of the causes of SCLAP. Among the 70 cases of metastatic adenocarcinoma, 49 presented with left-sided SCLAP, 20 with right-sided and one case with bilateral enlarged supraclavicular nodes. The most common site for primary left-sided SCLAP was the lung in 12 (24.5%) cases, followed by the gallbladder in 11 (22.5%), and the breast in 7 (14.3%) cases. The stomach was seen as primary in only 4 (8.1%) cases, while the duodenum was identified as primary in 3 (6.1%) cases. In our study, the primary site was unknown in 10 patients (20.4%) of left side supraclavicular lymphadenopathy. The liver and oral cavity were seen as primary in one case each. In patients with right-sided SCLAP and diagnosed with metastatic adenocarcinoma, the most common primary tumor was observed in the lung with 10 (50%), followed by the breast (n = 8, 40%). For right side lymphadenopathy, the gallbladder was identified as primary in one case, and in one case, the primary was not known. In metastatic squamous cell carcinoma, the most common primary site was the oral cavity, both for left- and right-sided SCLAP, seen in 22(78.6%) cases, followed by the lung in two (7.1%) cases. The cervix, esophagus, and Marjolin’s ulcer were observed in one case each, and the primary site was unknown in one case (Table 3).

**Table 1:** Distribution of malignant and non-malignant cases as per the demographic profile

	**Malignant (n=130)**	**Non-Malignant (n=90)**	**p-value**
**Gender** Male Female	60 70	28 62	0.02
**Laterality** Left side Right Side Bilateral	84 45 01	39 50 01	0.002
**Age** <40 years >40 years	34 96	69 21	< 0.00001

**Table 2:** Distribution of the cases as per the broad diagnostic category and laterality (N=220)

**Diagnosis**	**Right side (%)**	**Left sided (%)**	**Bilateral (%)**	**No. of cases (%)**
Metastasis	40 (18)	79 (36)	01(0.5)	120 (54.5)
Tuberculous lymphadenitis	37 (16.8)	19 (8.6)	01 (0.5)	57 (25.9)
Reactive lymphadenitis	5 (2.3)	6 (2.7)		11 (5)
Acute suppurative lymphadenitis	8. (3.6)	14 (6.4)		22 (10)
Lymphoma	05 (2.3)	05 (2.3)		10 (4.6)
Total	95 (43.3)	123 (56)	02 (0.5)	220 (100)

**Table 3:** Distribution of metastatic cases according to the Histological diagnosis, primary site and laterality

**Primary site**	**Right side**	**Left side**	**Bilateral**	**Total 120 cases (%)**
**Lung** Adenocarcinoma Squamous cell carcinoma Small cell carcinoma	10 2 1	12 0 1	1 0 0	**27 (22.5)**
**Breast** Infiltrating ductal carcinoma – NOS	8	7		15 (12.5)
**Gall bladder** Adenocarcinoma	1	11		12 (10)
**Stomach** Adenocarcinoma	0	4		4 (3.3)
**Duodenum** Adenocarcinoma	0	3		3 (2.5)
**Oral cavity** Squamous Cell Carcinoma Adenocarcinoma- NOS	11 0	11 1		**23 (19.2)**
**Testis** Germ Cell Tumor	0	3		3 (2.5)
**Urinary Bladder** Urothelial carcinoma	0	1		1 (0.83)
**Liver** Adenocarcinoma	0	1		1 (0.83)
**Esophagus** Squamous cell carcinoma	1	0		1 (0.83)
**Cervix** Squamous Cell carcinoma	0	1		1 (0.83)
**Skin** Squamous cell carcinoma	1	0		1 (0.83)
**Unknown primary** Adenocarcinoma Squamous Cell carcinoma	1 0	10 1		12 (10)
Poorly differentiated/metastatic epithelial malignancy	4	12		16 (13.3)
Total				**120 (100)**

## DISCUSSION

Supraclavicular nodes (SCLN) are located in the thoracic duct drainage path, which explains why abdominal neoplasms metastasize to this group of lymph nodes. There has been knowledge of this since the 19th century, and it has even led to the term Virchow’s or Virchow-Troisier node to refer to left SCLN metastasis from the gastric primaries (1,5). In most studies, SCLN (especially the left SCLN) is affected by abdominal or pelvic cancer (6,7). In addition, metastatic tumors of the chest, abdomen, and pelvis have also been reported in SCLN (8). Often, enlargement of a supraclavicular lymph node signals the onset of malignancy underlying the node, which requires prompt action to prevent further dissemination since most of them are malignant in origin. FNAC is a quick, painless, and minimally invasive procedure widely used to diagnose supraclavicular lymphadenopathy, particularly in cases of metastasis. In the present study, metastatic tumors were the most common cause of supraclavicular lymphadenopathy (54.5%), consistent with the findings of other studies by Adhikari et al., Gupta et al., and Kanetkar et al. (9–11). Another study conducted by Raja et al. found that out of 320 cases of SCLN, 33% were reactive in nature, and 40.4% of cases were diagnosed as non-malignant, encompassing granulomatous, reactive, and acute inflammatory lesions (12) (Table 4). However, the predominant histopathological subtypes of tumors affecting the supraclavicular region differed based on the findings of the present study. Giridharan et al. have found that most histological diagnoses were squamous cell carcinoma, followed by lymphoma, adenocarcinoma, and undifferentiated carcinoma (13). Fernández Aceñero et al. reported adenocarcinoma was the most common type of malignancy that metastasized to the SCLN, followed by squamous cell carcinoma, as observed in our study (14). Our findings were further supported by Gupta et al. who reported metastasis in 64% of the cases (10). In this study, we assessed the origin of primary tumors and found that the primary sites were the lungs, breast, oral cavity, testis, urinary bladder, hepatobiliary system, gastrointestinal tract, cervix, and skin. Among these primary sites, the lung was the most common (21%), followed by the oral cavity (17.6%) and breast (11.5%). In a study by Ismi et al, the lung was the most common primary site of origin, which further supports our findings (15). Breast cancer that metastasizes to the supraclavicular node is rare (2.3% - 4.3%) (16). However, in our study, it was found in 11.5%. We found only four cases (3%) originating from the stomach primary, as supported by Fernández Aceñero et al. reporting that 2% of cases metastasized from the stomach (14). Although all four cases of primary stomach presented with left-sided SCLAP, the most common primary site of metastasis to the left side was the lung, followed by the gallbladder and oral cavity. A study found that genitourinary neoplasms also contribute to SCLN metastasis (10). Tuberculous lymphadenitis was the second most common diagnosis in our setup on both sides, comprising 25.9 % of cases, followed by nonspecific acute inflammatory pathology in 9.5% of cases. Our findings are in concordance with those of Adhikari et al. (9) and Yadav et al. (17) who reported tuberculosis in 45% and 44.7 % of cases respectively. The supraclavicular area, extending from the infraclavicular sites to the central venous system, is the last common pathway in the lymphatic system. The lymph nodes of the common iliac and para-aortic arteries drain into the thoracic duct, which communicates with the systemic venous system at the junction of the left subclavian and internal jugular veins. The location of supraclavicular metastasis differs according to the distant metastatic site. A left supraclavicular lymph node (Virchow’s) is regarded as the last common pathway of infra-diaphragmatic sites (18). Therefore, infra-diaphragmatic tumors from the abdominal cavity mostly metastasize to the left supraclavicular fossa, while supra-diaphragmatic tumors from the thoracic cavity can metastasize to both the left and right (2). It is possible that the left supraclavicular lymph nodes are involved in metastasis of gastric carcinoma (Virchow’s node) (19).

**Table 4:** Comparison of the present study with the other studies

**Authors**	**Total Cases**	**Diagnosis (%)**				
		**Tuberculosis**	**Reactive** **Lymphadenitis**	**Acute Suppurative** **Lymphadenitis**	**Lymphoma**	**Metastatic Malignancies with most common primary site of origin**
Adhikari et al. (9)	149	41.6%	8.7%	-	4.8%	44.9% Primary site: lung (43.3%)
Fernández Aceñero et al. (14)	95	4.2%	16.8%	-	11.5%	60% Primary site: lung (38%)
Raja et al. (12)	320	9%	33%	14%	-	29% Primary site: head and neck region (16%)
Gupta et al. (10)	200	13.5%	10%	-	4.7%	64% Primary site: lung (22%)
Yadav et al. (17)	230	44.7%	7.4%	-	2.6%	30% Primary site not mentioned.
Present study	220	25.9%	5%	10 %	4.6%	54.5% Primary site Lung (22.5%)

A diagnosis of enlarged lymph nodes in the supraclavicular region should include evaluation of the digestive tract, tracheobronchial tree, breast, genitourinary tract, and thyroid gland as the primary sites. As in our study, all the infradiaphragmatic tumors, involving the gastric, urinary bladder cervix, and testis, metastasized to the left supraclavicular region. In contrast, 13 out of 27 cases of lung cancer metastasized to the left and 13 cases to the right SCL, and one case metastasized to both sides. As most supraclavicular tumors are malignant in origin, they need to be evaluated without delay, as patients may present with this symptom only. The suspicion of malignancy should increase if the patient is > 40 years old and presents with left-sided SCLAP. Lymph node aspiration plays a key role in the diagnosis of malignant lymphadenopathy. The clinical and radiological findings should be examined in conjunction with FNAC.

The findings underscore the evolving clinical significance of Virchow’s node in the context of changing cancer epidemiology. The data reaffirm the value of FNAC as a rapid and minimally invasive diagnostic tool that aids in timely detection and management of both malignant and benign conditions.

### Study Limitations

This study has certain limitations. Being retrospective and conducted at a single tertiary care center, the findings may not be generalizable to other populations or settings. Additionally, the lack of histopathological confirmation for FNAC diagnoses could affect the accuracy and reliability of the results. Future prospective studies incorporating histological correlation and multi-institutional data could provide a more comprehensive understanding of the diagnostic spectrum of supraclavicular lymphadenopathy.

## CONCLUSION

This study emphasizes the changing diagnostic spectrum of supraclavicular lymphadenopathy, and particularly the low incidence of gastric cancer as a primary site in our geographic region. The predominance of metastatic adenocarcinoma, especially from the lung, gall bladder and breast, underscores the need for tailored diagnostic and therapeutic approaches based on regional cancer epidemiology. FNAC has proven to be an invaluable tool in identifying malignant and benign etiologies, facilitating early interventions and improved patient outcomes.

## Conflict of Interest

The authors have no conflicts of interest to declare.
